# Proton Pump Inhibitor Induced Hypomagnesemia Causing Seizures: A Rare Adverse Effect of a Commonly Used Medication

**DOI:** 10.7759/cureus.64044

**Published:** 2024-07-07

**Authors:** Manojkumar Krishnan, Hiruni Fernando, Hassan H Mohammed, Neranjana Vithanage

**Affiliations:** 1 Medicine, Sri Jayawardenepura General Hospital, Colombo, LKA; 2 Medicine, Postgraduate Institute of Colombo, Colombo, LKA; 3 Chemical Pathology, Sri Jayawardenepura General Hospital, Colombo, LKA

**Keywords:** intestinal magnesium loss, etiology of hypomagnesemia, proton pump inhibitors (ppi), hypomagnesemia, serum magnesium

## Abstract

Hypomagnesemia is defined as serum magnesium levels less than 0.7mmol/L and can result in a plethora of symptoms ranging from mild gastrointestinal symptoms to serious conditions such as cardiac arrhythmias and neurological complications. When considering the etiological factors, drug-induced hypomagnesemia is highlighted because commonly used medications such as proton pump inhibitors (PPIs), aminoglycoside antibiotics, and loop and thiazide diuretics can cause low magnesium levels.

A 49-year-old male presented to the emergency department with severe vomiting worsening over three days complicated with generalized tonic-clonic seizures. He was an averagely built male, a non-smoker, and a non-alcoholic with no significant co-morbidities. He had a history of chronic over-the-counter pantoprazole intake over the last one year, and apart from that, his medication history was unremarkable. Initial investigations revealed severe hypomagnesemia (with serum magnesium level of 0.1mmol/L), with marginal hypokalemia, hypophosphatemia, and hypocalcemia. Following the initial resuscitation and magnesium supplementation, the patient’s clinical condition significantly improved. Due to the lack of proper knowledge, the patient continued to take omeprazole 20mg tablets after discharge, and up until proper education and total cessation of PPIs, marginal hypomagnesemia, with serum magnesium level of 0.5mmol/L, persisted without any significant overt clinical manifestations.

In this case report, we intend to highlight the importance of assessing for all possible electrolyte abnormalities in a patient presenting with neurological symptoms, relevance of taking a thorough drug history including all undocumented over-the-counter medications, and importance of patient education in the prevention of further episodes.

## Introduction

Magnesium is the fourth most commonly found cation in the body [1]. Usually, in the clinical practice, in the initial evaluation of a patient, magnesium levels are not routinely tested and thus are often referred as the “forgotten cation” in the literature [2].

Even though hypomagnesemia is defined as serum magnesium levels less than 0.7mmol/L, clinical features usually tend to occur when magnesium levels fall below 0.5mmol/L [3]. While severe hypomagnesemia causes overt neurological and cardiac symptoms, even mild hypomagnesemia is associated with long-term complications such as increased risk of diabetes mellitus, osteoporosis, and cardiovascular disease [4].

Hypomagnesemia can result from decreased intake, increased renal and gastrointestinal losses, as an adverse effect of medication, and due to redistribution from extracellular space into intracellular space [3]. The commonly used medications that can cause hypomagnesemia include proton pump inhibitors (PPIs), and loop and thiazide diuretics [3]. Specially, PPIs are available as over-the-counter medications, and long-term PPI use among patients is quite a common finding in day-to-day clinical practice. Even though these are commonly used medications, since magnesium levels are not routinely checked, mild hypomagnesemia can persist unnoticed until it progresses to severe hypomagnesemia with concurrent occurrence of other exacerbating factors, and the patient can end up with serious complications such as seizures and arrhythmias.

## Case presentation

A 49-year-old previously healthy male patient presented to the hospital with severe vomiting persistently worsening over the last three days. Initially, he had around two to three vomiting episodes per day, which worsened over time, and at admission, he was having around eight to 10 vomiting episodes per day. He did not complain of having any preceding or ongoing fever, abdominal pain, diarrhea, headache, or urinary symptoms. The vomitus was not blood or bile stained and contained undigested food particles.

On the day of the admission, he developed drowsiness and worsening altered level of consciousness. Just prior to the admission, he had also developed a seizure episode where he had generalized increased tonicity of all four limbs with few clonic movements lasting for around 2 minutes, with associated loss of consciousness, urinary incontinence, and post ictal drowsiness.

He was a non-alcoholic and a non-smoker and he did not have a significant past medical or surgical history. There was no history of previous seizure episodes as well. He did not have any allergies, and he previously had not been screened for any non-communicable diseases. He had been experiencing mild dyspeptic symptoms from time to time and had taken over-the-counter oral pantoprazole 40mg tablets over the course of the last one year preceding the admission. Apart from that, he had not been on any other regular medications.

On examination, he was afebrile with a Glasgow coma scale (GCS) score of 14 out of 15. He was drowsy and disoriented in relation to time and place and appeared to be mildly dehydrated. He did not have any focal neurological deficits, and neurological examination including fundus examination was normal with no cerebellar signs. His abdomen was soft and non-tender with no organomegaly. He had vesicular breathing with no added lung sounds and had normal respiratory rate. He had a mild tachycardia of 102 beats per minute with a blood pressure of 130/80, and the rest of his cardiovascular examination was unremarkable.

Upon admission to the hospital, a non-contrast computed tomography (NCCT) of the brain was performed and capillary blood sugar levels were screened at the emergency treatment unit (ETU), which did not reveal any intracranial hemorrhages or hypoglycemia (capillary blood sugar level was 92mg/dL at presentation). Initial blood investigations performed are summarized in Table 1.

**Table 1:** Summary of investigations WBC, white blood cells; CRP, C-reactive protein; ESR, erythrocyte sedimentation rate; AST, aspartate transaminase; ALT, alanine transaminase; ALP, alkaline phosphatase

	Value	Unit	Reference range
WBC	11.5	×10^3^/µL	4–11
Neutrophils	7.6	×10^3^/µL	2–7
Lymphocytes	2.8	×10^3^/µL	0.8–4
Hemoglobin	12.1	g/dL	11–16
Platelets	338	×10^3^/µL	150–450
CRP	<2	mg/L	<6
Sodium	140	mmol/L	136–145
Potassium	3.3	mmol/L	3.5–5.3
Chloride	98.8	mmol/L	97–111
Magnesium	0.1	mmol/L	0.7–1
Adjusted calcium	8.2	mg/dL	8.6–10
Inorganic phosphorus	2.1	mg/dL	2.7–4.5
Serum creatinine	65	µmol/L	45–90
ESR	19	mm/hour	0–30
AST	38	U/L	0–37
ALT	36	U/L	7–35
ALP	66	U/L	30–120
Total bilirubin	0.6	mg/dL	0.2–1.1
Albumin	3.8	g/dL	3.7–5
Serum amylase	120	U/L	40–140

In the initial workup, the most striking abnormality detected was severe hypomagnesemia along with mild hypokalemia, hypocalcemia, and hypophosphatemia. Arterial blood gas (ABG) performed on admission revealed metabolic alkalosis with a pH value of 7.49 with a bicarbonate value of 32.5mmol/L. Serial Electrocardiograms (ECGs) were also performed, but they did not reveal any abnormalities.

The patient was initiated on IV (intravenous) ondansetron 4mg and IV ranitidine 50mg and was hydrated with IV 0.9% NaCl with input and urine output monitoring. He was administered 1g of IV 20% magnesium sulphate over 15 minutes, and repeated doses were given three times until his altered mental status was improved. Since only mild hypokalemia and hypocalcemia were present, supplementation with oral potassium chloride and calcium carbonate was also started.

Following the initial management, his vomiting reduced with improvement in the hydration level and the repeat ABG was also normal. After completing three cycles of IV magnesium sulphate over the course of one day, his serum magnesium levels were retested, which showed an increased to 0.5mmol/L. To assess the etiology for severe vomiting and dyspeptic symptoms, an upper gastrointestinal endoscopy (UGIE) and an ultrasound scan (USS) of the abdomen were also performed, both of which turned out to be normal, and tests for Helicobacter pylori yielded to be negative as well. To rule out other causes for the seizure episode, a magnetic resonance imaging (MRI) scan of the brain was also performed, which did not reveal any space-occupying lesions.

With hydration and antiemetics, the patient’s condition drastically improved and he was discharged with oral supplementations of magnesium, calcium, potassium, and vitamin D and was planned to be reviewed at the clinic level with repeat electrolytes reports. The patient was advised to refrain from taking over-the-counter pantoprazole and was prescribed oral ranitidine for his dyspeptic symptoms.

The patient was reviewed up in the medical clinic in one week and then again in three weeks following the discharge with repeat electrolyte counts. Despite the patient being utterly asymptomatic with normal potassium, calcium, and sodium levels, the serum magnesium was persistently around 0.5mmol/L. Even though the cause for hypomagnesemia was postulated to be occurring secondary to prolonged pantoprazole intake, worsened by the recent onset vomiting, to exclude any renal losses of magnesium, urine magnesium levels and urine creatinine were also tested, which were 0.02mg/dL and 0.8mg/dL, respectively. Fractional excretion of magnesium was calculated for serum magnesium level of 0.5mg/dL, which revealed to be 3.68% excluding any renal losses of magnesium.

Upon further inquiry, it was revealed that the patient has continued taking omeprazole 20mg under a different brand name. The patient was thoroughly educated regarding the causative nature of hypomagnesemia with any PPIs and was advised to refrain from taking any over-the-counter medications.

The patient was again reviewed at the clinic in one month period with repeat serum electrolyte values, and following the total withdrawal of PPIs, it was noted that his magnesium levels had returned to a normal value of 0.9mmol/L.

## Discussion

Magnesium is an essential cation that is a key component in many metabolic pathways and is the fourth most commonly found cation in the body. Approximately 99% of body’s magnesium is in the intracellular space, with 50%-65% of it residing in bone. Out of the 1% extracellular magnesium, 70% is in the ionized active form [5]. When serum magnesium levels fall below the normal range of 1.7 to 2.3 mg/dL (1.4 to 2.1 mEq/L [0.7 to 1.05 mmol/L]), it is known as hypomagnesemia [2]. Oftentimes, hypomagnesemia has been known to occur in conjunction with other electrolyte abnormalities such as hypocalcemia and hypokalemia: hypocalcemia due to reduction in the release of parathyroid hormone and hypokalemia due to increased urinary excretion of potassium due to inhibition of renal medullary potassium channels [3]. In our case as well, marginally low levels of hypomagnesemia and hypocalcemia were noted.

Hypomagnesemia can be due to decreased intake, increased renal and gastrointestinal losses, as an adverse effect of medication, and due to redistribution from the extracellular space into the intracellular space [3]. The commonly used medications that can lead to hypomagnesemia are loop and thiazide diuretics, PPIs, and aminoglycoside antibiotics. Apart from those commonly used medications, other medicines such as digitalis, cisplatin, and cyclosporin can also lead to hypomagnesemia [3].

Even though hypomagnesemia is defined as magnesium levels below 0.7mmol/L, clinical manifestations usually tend to occur once the magnesium levels fall below 0.5mmol/L. These include neurological symptoms such as tremors, tetany, delirium, and convulsions, and cardiovascular symptoms such as fatal arrhythmias. Other contributory factors for fatal arrhythmias are concurrent hypokalemia and hypocalcemia that are known to occur alongside hypomagnesemia [6].

PPIs are thought to cause hypomagnesemia by reducing the active magnesium absorption that occurs via transient receptor potential melastatin 6 and 7 (TRPM6/7) [7]. Hypomagnesemia usually occurs when PPIs are taken over long periods of time, but in critically ill patients, it has been observed following a short course of PPIs as well [1].

Generally, in clinical practice, all it takes is a good history to identify the cause for hypomagnesemia. However, if an etiology cannot be identified or if there is evidence to suspect urinary magnesium loss as well, measuring 24-hour urinary magnesium excretion or fractional excretion of magnesium can help differentiate between gastrointestinal and renal losses [8].

In our patient, on the initial presentation, the causative factor for hypomagnesemia was postulated to be mainly due to severe vomiting and starvation over the preceding one week, which acted as an exacerbating factor to an already existing mild asymptomatic hypomagnesemia caused by prolonged use of PPIs. The persistent hypomagnesemia that only responded to total cessation of PPIs also supported this theory. Even though the patient was advised to refrain from taking PPIs, the fact that the patient continued to take omeprazole instead of the pantoprazole he previously used highlights the importance of proper patient education in scenarios like these. Oftentimes, the patients may not have a good understanding about how there can be multiple medications belonging to the same drug class and that the side effects can be shared between different drugs of the same drug class. Hence, it is important to provide proper patient instructions in situations like these.

Management of hypomagnesemia depends on the severity of symptoms. Severe neurological and cardiac symptoms with associated hemodynamic instability warrants IV magnesium sulphate therapy, where 1-2g of IV magnesium sulphate should be given as an infusion over 2 to 15 minutes. Oral magnesium sulphate is only given to asymptomatic patients with mild hypomagnesemia [2].

## Conclusions

In a patient presenting with neurological symptoms such as convulsions, it is important to actively look for all likely electrolyte abnormalities, including magnesium levels. Undiagnosed hypomagnesemia can result in serious and sometimes fatal consequences such as repeated convulsions and fatal arrhythmias. Once the hypomagnesemia has been identified, it is imperative to identify the underlying cause and correct it. Sometimes, long-term administration of commonly used over-the-counter medications such as PPIs can be missed during the initial history taking if not actively sought out. Symptomatic management of the complications should be done in the acute stage along with addressing the underlying cause and magnesium supplementation. It is also important to advise the patient regarding the association between hypomagnesemia and prolonged PPI use to avoid further episodes.
